# A Cohort Study in Intensive Care Units: Health Decisions Related to Blood Transfusion during the COVID-19 Pandemic

**DOI:** 10.3390/jcm11154396

**Published:** 2022-07-28

**Authors:** Raúl Juárez-Vela, José Antonio García-Erce, Vicente Gea-Caballero, Regina Ruiz de Viñaspre-Hernandez, José Ángel Santos-Sánchez, Juan Luis Sánchez-González, Eva María Andrés-Esteban, Michał Czapla, Clara Isabel Tejada, Kapil Laxman Nanwani-Nanwani, Ainhoa Serrano-Lázaro, Manuel Quintana-Díaz

**Affiliations:** 1 Doctoral Program in Medicine and Surgery, Faculty of Medicine, Autonomous University of Madrid, 28049 Madrid, Spain; raul.juarez@unirioja.es (R.J.-V.); mquintanadiaz@gmail.com (M.Q.-D.); 2 Research Group in Care (GRUPAC), Faculty of Health Sciences, University of La Rioja, C/Duquesa Victoria 88, 26006 Logrono, Spain; reruizde@unirioja.es (R.R.d.V.-H.); michal.czapla@umw.edu.pl (M.C.); clara-isabel.tejada@unirioja.es (C.I.T.); 3 Blood Management Patient Group, Research Institute Idi-Paz, 28046 Madrid, Spain; vagea@universidadviu.com (V.G.-C.); e.andres@live.com (E.M.A.-E.); kapilnanwani@gmail.com (K.L.N.-N.); aserranolazaro@gmail.com (A.S.-L.); 4 Bank of Blood and Tissue of Navarra, Government of Navarra, 31008 Pamplona, Spain; 5 Faculty of Health Sciences, Valencian International University, 46002 Valencia, Spain; 6 Faculty of Medicine, University of Salamanca, 37008 Salamanca, Spain; jasalao@usal.es; 7 Salamanca Hospital Complex, 37008 Salamanca, Spain; 8 Faculty of Nursing and Physiotherapy, University of Salamanca, 37008 Salamanca, Spain; juanluissanchez@usal.es; 9 Department of Business Economics and Applied Economy, Faculty of Legal and Economic Sciences, University Rey Juan Carlos, 28032 Madrid, Spain; 10 Department of Emergency Medical Service, Wroclaw Medical University, 51-616 Wroclaw, Poland; 11 Institute of Heart Diseases, University Hospital, 50-566 Wroclaw, Poland; 12 La Paz Hospital Intensive Care Unit, 28046 Madrid, Spain; 13 Intensive Care Unit, Hospital Clinico de Valencia, 46010 Valencia, Spain

**Keywords:** blood transfusion, patient blood management, coronavirus infections, hemorrhage, critical care

## Abstract

Critically ill polytrauma patients with hemorrhage require a rapid assessment to initiate hemostatic resuscitation in the shortest possible time with the activation of a massive transfusion or a critical hemorrhage management protocol. The hospital reality experienced during the COVID-19 pandemic in all countries was critical, as it was in Spain; according to the data published daily by the Ministry of Health on its website, during the period of this study, the occupancy rate of intensive care units (ICUs) by patients diagnosed with the novel coronavirus disease (COVID-19) rose to 23.09% in Spain, even reaching 45.23% at the end of January 2021. We aimed to analyze the changes observed during the severe acute respiratory syndrome coronavirus 2 (SARS-CoV-2) pandemic period regarding the effectiveness of Spanish ICUs in terms of mortality reduction. We present a cross-sectional study that compares two cohorts of patients admitted to ICUs across all autonomous communities of Spain with a diagnosis of polytrauma. Results: Only age was slightly higher at admission during the first wave of the pandemic (47.74 ± 18.65 vs. 41.42 ± 18.82 years, *p* = 0.014). The transfusion rate during the pandemic increased by 10.4% compared to the previous stage (*p* = 0.058). Regarding hemostatic components, the use of tranexamic acid increased from 1.8% to 10.7% and fibrinogen concentrates from 0.9% to 1.9%. In the case of prothrombin complex concentrates, although there was a slight increase in their use, there were no significant differences during the pandemic compared to the previous period. Conclusion: Mortality showed no difference before and during the pandemic, despite the observed change in the transfusion policy. In summary, the immediate and global implementation of patient blood management (PBM) based on clinical transfusion algorithms should be mandatory in all hospitals in our country.

## 1. Introduction

Today, the most frequent cause of mortality in young adults is trauma, causing more than five million deaths per year worldwide [1,2]. The main cause of mortality in trauma patients is associated with massive hemorrhage [3].

Several studies have concluded that the mortality rate in polytrauma patients could be reduced with a multidisciplinary approach that would allow a specific diagnosis to be made in order to initiate early treatment incorporating resuscitation techniques, rapid and concise surgical management, or infusion of hemocomponents, crystalloids, and goal-directed early hemostatic reconstitution [1,4,5].

Critically ill polytrauma patients with hemorrhage require rapid assessment to initiate hemostatic resuscitation as fast as possible. The decision regarding the transfusion of concentrates has changed over time. Until a few years ago, it was expected that an increase in hemoglobin could increase the oxygen transport capacity of the blood and regenerate tissues.

Currently, in most hospitals in Spain, the effectiveness of care in intensive care units (ICUs) is usually evaluated using mortality indicators, often adjusted by the clinical severity of the admitted patient. Since then, important changes in the management of bleeding patients have occurred [6,7,8], which have been shown to improve the prognosis of such patients and are therefore being incorporated into transfusion guidelines.

An example is the administration of tranexamic acid, the use of coagulation point-of-care, the early use of plasma hemoderivatives (fibrinogens and prothrombin complex concentrates), and the use of combined blood products in transfusion ratios close to 1:1:1 (red cell concentrates/platelet concentrates/fresh frozen plasma). Some authors have introduced the term “cost of critical patient management” to express the efficiency of ICUs, but there is no consensus on assessing the quality of care received by polytrauma patients [9,10]. The hospital reality experienced during the COVID-19 pandemic in all countries was critical, as it was in Spain; according to the data published daily by the Ministry of Health [11], during the period of this study, the occupancy rate of ICUs by patients diagnosed with COVID-19 rose to 23.09% in Spain, even reaching 45.23% by the end of January 2021 (data eliminating the first wave).

During this period, healthcare professionals were under enormous pressure due to COVID-19, which could undermine the care that would be required by a patient with any other type of illness or injury admitted to these ICUs [12]. For all of these reasons, it is essential to know and describe the type and quality of care received by patients admitted to ICUs for polytrauma in our country, how they were treated and their prognosis, and to compare these figures with other series published before the pandemic.

To evaluate transfusion adequacy, we relied on the hemoglobin (Hb) levels presented by each patient, analyzing whether it corresponds to the established standards. Following the recommendations made by different guides, such as the American Association of Blood Banks, we considered the following parameters to determine whether transfusion presents an adequate indication: patients without any type of previous heart disease: Hb ≤ 8 g/dL; patients with previous heart disease: Hb ≤ 9 g/dL.

We aimed, as our main objective, to analyze the changes observed during the SARS-CoV-2 pandemic period regarding the effectiveness of Spanish ICUs in terms of blood transfusion.

## 2. Materials and Methods

### 2.1. Design

This research comprised a cohort descriptive study, comparing two cohorts of patients admitted to ICUs in all the autonomous communities of Spain (see Table 1) with a diagnosis of polytrauma (a person who suffers a multiple trauma with the involvement of several anatomical regions or organs). The first cohort took place over a period of one month, from 1 to 30 November 2019, and the second cohort took place over a period of six months, specifically from 1 May to 30 October 2021.

Due to the restriction of movement during the pandemic period, the second cohort took place over a longer period because there were fewer polytraumatized patients with a lower rate of admission.

### 2.2. Variables

The following information was collected through electronic means. Epidemiological variables: Age, sex, date of trauma and admission to the ICU, intentionality, mechanism and type of trauma, previous intake of antiplatelet or anticoagulant drugs, and previous comorbidities.

Registration of traumatic injuries according to the Abbreviated Injury Scale (AIS) [13,14] and the estimation of different severity indices such as the ISS scale, TRISS [6] the revised trauma scale, and other more general scales used in intensive care units, such as the SOFA (Sequential Organ Failure Assessment Score), APACHEII (Acute Physiology and Chronic Health disease Classification System), and SAPS II (Simplified Acute Physiology Score) [15].

All of the applied scales allowed us to reliably measure severity and have previously been validated for use in our setting.

### 2.3. Data Collection

Data collection was carried out using the creation of an electronic data collection notebook (CRDe) so as to obtain the medical outcomes, which will allow physicians to make better decisions in the future. All of the ICUs of the ACs involved could enter their data directly. The use and registration of medical variables was achieved by each center manager, who could access this CRDe using a username and password, but they only had access to view and modify the data for their center. Each patient was assigned an anonymized code, which was kept by the researcher at each center and made it impossible to recognize the patient. The information was treated confidentially and anonymously, since it was dissociated data, following the Data Protection Regulation (EU) 2016/679 of the European Parliament and the Spanish Law.

### 2.4. Statistical Analysis

Quantitative variables are described using the mean and standard deviation or median and interquartile range according to the type of distribution. The normality of the distribution was analyzed using the Shapiro–Wilks test. Categorical variables are expressed via their frequency distributions and percentages.

To analyze the differences between the categorical variables of the two cohorts, a chi-square test was used, or Fisher’s exact test if the hypotheses of applicability of the former were not met. If the variables were quantitative, we used Student’s *t*-test or the Mann–Whitney *U*-test according to the normality criteria.

Statistical analysis was performed with the STATA/SE v.21.0 program (College Station, TX, USA), with any value of *p* < 0.05 being considered statistically significant.

## 3. Results

A total of 111 ICUs from public hospitals participated in the study (Table S1).

**Figure 1:**
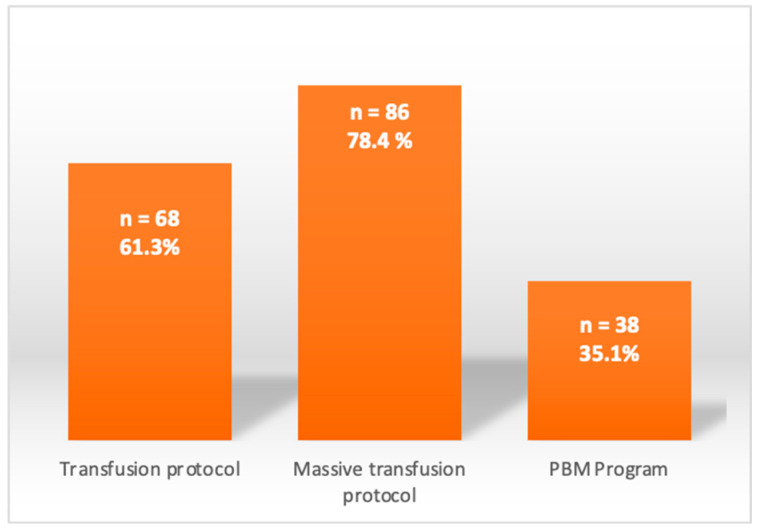
Transfusion policies of the hospitals involved. Note. PBM = Patient Blood Management.

The availability of ICU beds before the pandemic was 23.41 ± 10.31 beds, with a range of 6–48 beds, thus collecting information on different size capacities and hospital levels. Only 10% were trauma ICUs; the remaining participating ICUs were multipurpose. Figure 1 shows the transfusion policies of the study hospitals regarding the management of patients’ bleeding: 61.3% of the transfusing policies were transfusion protocols (massive transfusion protocols or patient blood management programs), 78.4% were massive transfusion protocols (referring to rapid administration of large amounts of blood products in fixed ratios, usually 1:1:1 red blood cell concentrates/fresh frozen plasma/platelets for the management of hemorrhagic shock), and only 35.1% were patient blood management programs (multidisciplinary evidence-based approach to optimize the care of patients who need a blood transfusion).

Table 1 shows the demographic characteristics of the polytrauma patients admitted to the ICU before and during the COVID-19 pandemic. There were no differences in the severity of the patients’ condition upon admission to the ICU in any of the scales used. Only age was slightly higher at admission during the COVID-19 pandemic, with a mean value of 47.74 ± 18.65 versus 41.42 ± 18.82 years (*p* = 0.014). The overall transfusion rate during the pandemic increased by 10.4% compared to the previous stage (*p* = 0.058). The rate of 24-h exitus almost tripled during the pandemic (*p* = 0.051).

**Figure 2:**
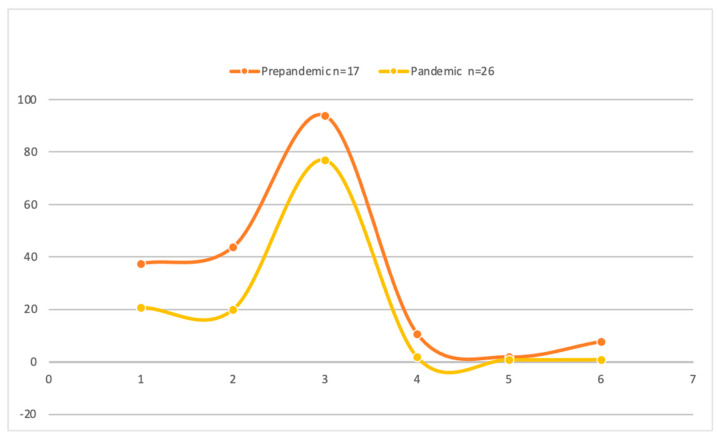
Number of blood and hemostatic components in both periods.

In Figure 2, we can observe a significant change in the blood components used in transfusions in both periods considered in this study. In the case of fresh frozen plasma, it decreased by 16.7% (*p* = 0.007); in the case of platelets, it decreased by 23.8% (*p* < 0.001); and in relation to red cell blood concentrates (RBCs), the reduction was 16.9% (*p* < 0.001). In the first period, a mean of two red cell blood concentrates (95% CI, 2.2–3.1) were transfused compared to 3.2 (95% CI, 2.0–4.19) (*p* < 0.001). Additionally, more fresh plasma was transfused, with an average of eight bags being administered (95% CI, 2.8–4.8) versus 2.4 bags (95% CI, 1.0–3.1) (*p* < 0.01). As for platelets, a mean of 3.6 pools (95% CI, 2.7–4.6) were administered versus 2.2 pools (95% CI, 0.15–4.24) (*p* < 0.01) with no significant difference in the amounts administered for any blood component. Related to hemostatic components, 1.8% were given tranexamic acid, 0.9% prothrombin complex concentrates, and 0.9% fibrinogen concentrates pre-pandemic. During the pandemic, the rate increased to 8.9% for tranexamic acid (*p* = 0.042), 1.0% for prothrombin complex concentrates (*p* = 0.864), and 6.9% for fibrinogen concentrates (*p* = 0.049).

## 4. Discussion

In this study, we analyzed the differences observed in blood transfusions and mortality during the SARS-CoV-2 pandemic period in Spanish ICUs. This study showed a transfusion rate in polytrauma patients admitted to the ICU of almost 15% in the period prior to the pandemic and 25% during this period, an increase that does not reach the limit of statistical significance but is very close to it. These figures, although a priori may attract attention, are similar to previous studies, such as that of Vicent et al. [16], carried out on patients admitted to the ICU for any cause, which reported a transfusion rate of around 26%. However, they are significantly lower than those obtained in other European studies, where a transfusion rate of 37% was estimated [17,18,19,20].

During COVID-19, blood donation centers in many parts of the world were suspended; the rate of donations decreased dramatically due to people being socially alienated, suffering from diseases or being in quarantine, as observed in the study by Al-Riyami et al. [20], where it was recorded that there was a decrease in blood donations of 70.6% in collection centers. Therefore, health policies focused on disease containment, while resources continued to be depleted beyond the demand required at the time [13,21], creating a supply/demand imbalance. For all of these reasons, it is essential to make this fact visible to anticipate similar situations and to make what is known as patient blood management (PBM) policies.

Based on the results of this study, the management of donations is especially important at the present time, marked by the continuity of COVID-19 [22,23]. The current pandemic situation caused by COVID-19 has created an environment in which the application of these practices is even more relevant [24], as blood is considered a scarce and very valuable resource with a limited storage time.

Related to the hemostatic components, the use of tranexamic acid increased from 1.8% to 10.7% and the fibrinogen concentrate from 0.9% to 1.9%. In the case of prothrombin complex concentrates, although there was a slight increase in use, there were no significant differences in the pandemic period compared to the previous period. In relation to the use of tranexamic acid, there are precedents confirming the reduction in non-immediate mortality, regardless of the severity of the patient [25]; it also allows a decrease in mortality as a result of traumatic hemorrhagic shock [26]. As for fibrinogen concentrates, the literature is clear on the relationship between low levels of fibrinogen, bleeding, and mortality, so their use and effectiveness were assessed regarding whether they are associated with the transfusion of red blood cell concentrates without the risk of thrombotic complications. However, the cost–benefit ratio of prohemostatics such as fibrinogen has been discussed [27], although the same applies to tranexamic acid, the recommendation for which is in favor of its use.

Concerning mortality, our results showed that it did not reach the limit of statistical significance but was very close to it. There were no significant differences between pre- and post-pandemic, despite a change in the transfusion policy, although we did report significantly higher mortality figures during the pandemic.

Mortality was measured at 24 h, so these data are not completely reliable and can be considered a serious limitation of this study. However, we believe it is worth clarifying, since it does allow us to study early mortality in trauma patients in the ICU.

In patients transfused in the first cohort, the use of blood components was well below the rate of patients receiving blood components during the pandemic (fresh frozen plasma and platelets). Regarding the transfusion of red cell blood concentrates during the pandemic period, we hypothesized two possibilities. One is that, during the pandemic, more transfusions were performed but with a lower volume being transfused, increasing the hemostatic drugs described in Figure 2. The second hypothesis was relative to the severity of the patient’s conditions upon admission to ICU, due to which 10.84% died in the first 24 h. These hypotheses have to be demonstrated with a cause-and-effect study design. It would be advisable to design new studies with a longer longitudinal follow-up to resolve this issue, which could also allow us to study in-depth the possible relationship between the type of protocols and products used, as well as the mortality associated with worse conditions and bringing about more transfusions.

PBM programs are a set of therapeutic strategies whose main objective is to improve patients’ conditions by focusing on preventing or avoiding the over-transfusion of blood, as this can lead to serious complications [22,23]. Our study was oriented toward studies carried out in the United States, where it has been shown that COVID-19 has had a clearly negative effect with regard to blood donations, which has led to the need to adapt policies against this decline [28]. Such new policies have been established through programs such as PBM. Different studies have introduced PBM educational initiatives to implement a system of periodic reports in which the rational use of blood components is described, as well as reports with experts in transfusion, all aspects indicating the need to establish PBM programs. These programs are mentioned in our study in light of the need to establish better bleeding management policies in trauma patients [29,30,31,32].

In general, the approach to trauma patients is complicated, since they present a complex condition in an unstable dynamic hemodynamic situation. The PBM approach cannot be underestimated because of the importance of teamwork, communication, high-level decision making, and documentation in dealing with critical situations [31].

Our study is related to other studies reporting a drop in blood during COVID-19 in ICUs of over 50%. Other studies, such as the first one carried out in China and the United States, have shown that 95% of ICU patients had anemia and that 85% of those were admitted to the ICU for seven days or more, which coincides with our study, in which a mean of 9.37 ± 12.03 days in the ICU [33,34,35,36,37].

Regarding the reason, there are many variables that must be taken into account and that must be contrasted with studies of cause and effect; however, according to our studies, as well as other published studies, our hypothesis is valid in that the main cause is a “transfusion trigger” rather than a physiologic need for blood. This means that there is not much adherence to clinical practice guidelines on transfusions, especially in ICUs—aspects that will be reduced with the establishment of a PBM [38,39], as has been recommended by the World Health Organization since 2010 and by the European Union since 2017.

## 5. Conclusions

It is important to monitor transfusions through public health decisions and the implementation of PBM programs. These programs and their implementation will optimize transfusion strategies in the face of a probable shortage of donations. Despite the current shortage of plasma and the decrease in reserves in hospitals and blood banks, the data showed a profound change in transfusion practices. In summary, the immediate and global implementation of patient blood management (PBM) should be mandatory in all hospitals in our country.

## 6. Limitations

The nature of this study does not allow us to establish cause–effect relationships, and only traumatized patients were considered. Another aspect that we have to consider as a limitation is that we epidemiologically compared two cohorts at two different times, which could have led to bias.

## Figures and Tables

**Table 1:** Demographic and clinical characteristics of the patients at admission to the ICU.

		Pre-COVID-19 Pandemic		During the COVID-19 Pandemic		*p*-Value
*N*		109		103		
Age (mean ± SD)		41.42 ± 18.82		47.74 ± 18.65		0.014
Sex	Men	93	85.32	80	77.67	0.151
	Women	16	14.68	23	22.33	
* SOFA-score (mean ± SD)		5.04 ± 3.46		4.68 ± 4.09		0.890
** APACHE II (mean ± SD)		15.40 ± 10.38		15.68 ± 10.85		0.567
*** SAPS II (mean ± SD)		36.25 ± 18.15		37.29 ± 20.07		0.635
Trauma Score Revised (mean ± SD)		6.95 ± 1.79		6.89 ± 1.78		0.403
^ ISS (mean ± SD)		23.13 ± 20.78		22.86 ± 19.86		0.461
^^ ICU stay (days; mean ± SD)		13.31 ± 20.53		9.37 ± 12.03		0.043
Mechanical ventilation	No	45	41.28	54	52.70	0.128
	Yes	64	58.72	49	47.30	
Extrarenal purification	No	107	98.17	100	97.14	0.652
	Yes	2	1.83	3	2.86	
Anticoagulation	No	102	94.44	99	96.12	0.568
	Yes	6	5.56	4	3.88	
Anti-aggregation	No	101	92.66	98	95.15	0.451
	Yes	8	7.34	5	4.85	
Exitus at 24 h	No	105	96.33	91	89.16	0.051
	Yes	4	3.67	12	10.84	
Blood components transfusion	No	92	84.40	77	74.76	0.058
	Yes	17	15.59	26	25.24	

SD = Standard Deviation. * SOFA Scale: Sequential Organ Failure Assessment Score. ** Apache: Acute Physiology and Chronic Health Disease Classification System; *** Simplified Acute Physiology Score II; ^ Injury Severity Score; ^^ ICU = intensive care unit.

## Data Availability

The datasets analyzed during the current study are available from the first author upon reasonable request.

## Supplementary Materials

The following supporting information can be downloaded at: https://www.mdpi.com/article/10.3390/jcm11154396/s1, Table S1. Participating Hospitals.
