# Supplementary material for: A Cohort Study in Intensive Care Units: Health Decisions Related to Blood Transfusion during the COVID-19 Pandemic
Source: J Clin Med. 2022 Jul 28;11(15):4396. doi: 10.3390/jcm11154396 (PMC9368991; doi:10.3390/jcm11154396)
Supplement: Supplementary file 1 [file jcm-11-04396-s001.zip › jcm-1788149-supplementary.pdf]

**Table S1. Participating Hospitals**

| Hospital | Region (CCAA) |
|---|---|
| Nacho Ortiz. Hospital Infanta Elena (Huelva) | Andalucía |
| Mª Antonia Lollano Zárate. Hospital Virgen de la Victoria de Málaga | Andalucía |
| Jesús Emilio Barrueco. Hospital General Universitario de Málaga | Andalucía |
| Marta Recio. Complejo Hospitalario de Jaen | Andalucia |
| Nora Palomo. Hospital Universitario Virgen del Rocío (Sevilla) | Andalucía |
| Antonio Gordillo. Hospital Puerta del Mar de Cadiz | Andalucía |
| Lorena Olivencia. Hospital Universitario Virgen de las Nieves (Granada) | Andalucía |
| Carmen de la Fuente Martos. Hospital Universitario Reina Sofía de Córdoba | Andalucía |
| Jose Garnacho/Gracia Gómez. Hospital Universitario Virgen Macarena | Andalucía |
| Isabel Sancho/Cristina Royo Vila. Hospital Miguel Servet | Aragon |
| Carlos Serón Arbeloa. Hospital San Jorge de Huesca | Aragón |
| Paula Ocabo. Hospital Clinico Universitario | Aragón |
| Gabriel Tirado. Hospital Royo Villanova | Aragón |
| Vitoria Rull. Mútua Maz | Aragón |
| Jose Mª Montón. Hospital Obispo Polanco (Teruel) | Aragón |
| Débora Fernández Ruiz. Hospital Universitario de Cabueñes | Asturias |
| Eva Ocampos Martinez. Hospital Valle del Nalón | Asturias |
| Ana Balán Mariñon. Hospital San Agustín de Aviles | Asturias |
| Jose Antonio Gonzalo/ Blanca Leoz Gordillo. Hospital Universitario Central de Asturias | Asturias |
| Patricia López Fajardo. Hospital Universitario Nuestra Señora de La Candelaria (Las Palmas) | Canarias |
| Silvia Puerto Corrales. Hospital Doctor José Molina Orosa (Lanzarote) | Canarias |
| Sergio Ruiz Santana. Hospital Universitario Dr Negrín (Las Palmas) | Canarias |
| Luis Ramos. H. General de La Palma. | Canarias |
| José Blanco López. Hospital Insular de Las Palmas | Canarias |
| Maria Luisa Mora. Hospital Universitario de Canarias | Canarias |
| Leandro Fajardo. Hospital General de Fuerteventura | Canarias |
| Ángeles Ballesteros. Hospital Universitario Marqués de Valdecilla | Cantabria |
| Alberto Silva. Hospital Universitario de Guadalajara | Castilla La Mancha |
| Miguel Angel Taberna. Hospital Ntra. Sra. del Prado de Talavera | Castilla La Mancha |
| Mª Luisa Gómez. Complejo Hospitalario de Ciudad Real | Castilla La Mancha |
| Luis Marina. Hospital Virgen de la Salud de Toledo | Castilla La Mancha |
| Isabel Murcia. Complejo Hospitalario de Albacete | Castilla La Mancha |
| Juan Carlos Pérez Llorens. Hospital Virgen de la Luz de Cuenca | Castilla La Mancha |
| Aaron Blandino. Hospital Santa Bárbara de Puertollano (Ciudad Real) | Castilla La Mancha |
| Ana María Domínguez Berrot. Complejo Asistencial Universitario de León | Castilla y León |
| Javier Gónzalez/Luis Alfonso Marcos Prieto/ Felix Martín Gónzalez Complejo Asistencia Universitario de Salamanca | Castilla y León |
| Leonor Nogales Martín. Hospital Clínico Universitario de Valladolid | Castilla y León |
| Rubén Herrán Monge. Hospital Universitario Río Hortega (Valladolid) | Castilla y León |
| Lorenzo Fernández. Hospital Nuestra Señora de Sonsoles (Ávila) | Castilla y León |

| | |
|---|---|
| María Gero Escapa/Paula de la Torre Vélez. Hospital Universitario de Burgos | Castilla y León |
| Diana Donaire. Complejo Hospitalario de Zamora | Castilla y León |
| Pablo Romero García. Complejo Asistencial de Soria. Hospital Santa Bárbara | Castilla y León |
| Teresita Alvarez/Mª Chanel Martínez Jiménez. Hospital El Bierzo de Ponferrada | Castilla y León |
| Xavier Soler. Clinica Teknon (Barcelona) | Cataluña |
| Luis Servia. Arnau de Vilanova (Lleida) | Cataluña |
| Belen Encina. Hospital Quiron (Barcelona) | Cataluña |
| Ana Parra. Hospital Sant Joan (Reus) | Cataluña |
| Baltasar Sánchez. Mutua de Terrassa | Cataluña |
| Mª Carmen Gilavert. Hospital Joan XXIIII (Tarragona) | Cataluña |
| Rosa Catalan. Hospital de Vic | Cataluña |
| Ricard Ferrer. Centro Médico Delfos | Cataluña |
| Carol Lorencio. Hospital Josep Trueta (Girona) | Cataluña |
| Estela Vals. Hospital Universitario Arnau Vilanova de Lleida | Cataluña |
| Juan Carlos Ruiz. Hospital Vall d'Hebron | Cataluña |
| África Lores/Virginia Alonso. Hospital de Bellvitge | Cataluña |
| Ana Ochagavía. Hospital Parc Taulí | Cataluña |
| Juan Carlos Yébenes. Hospital de Mataró | Cataluña |
| José Luís Jiménez. Hospital de Granollers | Cataluña |
| Pilar Marcos. Hospital Germans Trias i Pujol (Badalona) | Cataluña |
| Jose David Simón /Maria Ángeles Relucio Martínez. Hospital Vega Baixa de Orihuela | Comunidad Valenciana |
| Adoración Alcalá López/Diana Martínez López. Hospital General Universitario de Elche | Comunidad Valenciana |
| Francisco Ángel Jaime Sánchez. Hospital General Universitario Alicante | Comunidad Valenciana |
| Lorena Peiró Ferrando/Tatiana Villada Warrington. Hospital Universitario Sant Joan de Alicante | Comunidad Valenciana |
| Amparo Oliva. Hospital Comarcal de Vinaroz | Comunidad Valenciana |
| Fernando Sánchez Morán. Hospital Universitario La Plana de Villarreal | Comunidad Valenciana |
| Ainhoa Serrano Lázaro/Cristina Sanchis Piqueras. Hospital Clínico Universitario Valencia | Comunidad Valenciana |
| Maria Paz Fuset Cabanes/Karla Vacacela Córdova. Hospital Universitario La Fe Valencia | Comunidad Valenciana |
| Santiago Borrás Pallé/Luis García Ochando. Hospital Universitario Dr Peset Valencia | Comunidad Valenciana |
| Maria Desamparados Navarro Eduardo. Hospital Arnau de Vilanova Valencia | Comunidad Valenciana |
| Martín Parejo Montell/José Alberto López Baeza. Hospital de La Ribera Alzira | Comunidad Valenciana |
| Maria Luisa Micó Gómez/Sonia Gomar Vidal. Hospital de Manises | Comunidad Valenciana |
| Gemma Isabel Furió Mateo. Hospital General de Requena | Comunidad Valenciana |
| Maria Dolores García López/Nélida Cháfer Plasencia. Hospital Lluis Alcanyis de Xàtiva | Comunidad Valenciana |
| Manuel Palomo Navarro/Miguel Ángel García. Hospital de Sagunto | Comunidad Valenciana |

| | |
|---|---|
| Manuel Solera Suárez/Susana Isabel Gil García. Hospital Comarcal Francesc de Borja Gandía | Comunidad Valenciana |
| Elena Bisbal Andrés/Bárbara Vidal Tegedor. Hospital General Universitario Castellón | Comunidad Valenciana |
| Iker García Saenz. Hospital Donostia | Euskadi |
| Goiatz Balciscueta. Hospital Universitario de Araba | Euskadi |
| Mª José García Ramos. Hospital Universitario de Cáceres | Extremadura |
| Manuel Cidoncha. Hospital Don Benito Villanueva | Extremadura |
| Baldomero Jimenez. Hospital Universitario Infanta Cristina de Badajoz | Extremadura |
| Ana Isabel Tizón Varela. Complexo Hospitalario Universitario de Ourense | Galicia |
| Alejandra Pedreira. Complejo Hospitalario Universitario de Santiago | Galicia |
| Melinda Minia. Hospital Álvaro Cunqueiro de Vigo | Galicia |
| Maite Bouza. Hospital Universitario Lucus Augusti (Lugo) | Galicia |
| María Lourdes Cordero Lorenzana/Jorge Gamez Zapata/ Teresa Tabuyo Bello Complejo Hospitalario Universitario A Coruña | Galicia |
| Rocío Amézaga Menéndez. Hospital Universitari Son Espases | Illes Balears |
| Eduardo Antón Caraballo. Hospital de Manacor (Manacor) | Illes Balears |
| Gemma Rialp Cervera/María Romero Carratalá. Hospital Universitari Son Llàtzer (Palma) | Illes Balears |
| Susana Sánchez Pérez. Hospital Mateu Orfila (Menorca) | Illes Balears |
| Miguel Martín-Calpena Miranda. Hospital Comarcal d'Inca (Inca) | Illes Balears |
| Paz Merino de Cos. Hospital Can Misses (Ibiza) | Illes Balears |
| Concepción Pavía. Hospital San Pedro de Logroño | La Rioja |
| Aaron Blandino. Hospital Universitario Ramón y Cajal | Madrid |
| Ana Isabel Gónzalez/Sofia Rovirosa Bigot. Hospital Fundación de Alcorcón | Madrid |
| Daniel Ballesteros. Hospital Universitario Puerta de Hierro | Madrid |
| Carlos Velayos. Hospital Universitario de Fuenlabrada | Madrid |
| Victoria Enciso/Sofía García. Hospital Universitario del Henares | Madrid |
| Milagros Sancho. Hospital Universitario Gregorio Marañón | Madrid |
| Miguel Sánchez. Hospital Universitario Clínico San Carlos | Madrid |
| Alfonso Canabal. Hospital Universitario de La Princesa | Madrid |
| Jose Angel Lorente/Mercedes Rubio. Hospital Universitario de Getafe | Madrid |
| Judit Gutierrez. Hospital Universitario 12 de Octubre | Madrid |
| Maria Teresa Nieto. Hospital Universitario Infanta Elena | Madrid |
| Nieves Franco. Hospital Universitario de Mostoles | Madrid |
| Emilio Nevado. Hospital Universitario del Sureste (Parla) | Madrid |
| Eva Manteiga/Inma Colino. Hospital Infanta Cristina (Parla) | Madrid |
| Mª Ángeles Alonso. Hospital Universitario del Tajo | Madrid |
| Kapil Nanwani/Andrés Saravia. Hospital Universitario La Paz | Madrid |
| Manuel Párraga. Hospital General Universitario José María Morales Meseguer | Murcia |
| Aitor Ansotegui/Eva Regidor/Juan Angel Tihista Complejo Hospitalario de Navarra (Pamplona) | Navarra |

CCAA = Autonomous Region of Spain.
